# Serial Red Cell Distribution Width and Mortality After TAVR: An Observational Cohort Study

**DOI:** 10.3390/diagnostics16213659

**Published:** 2026-11-05

**Authors:** Zhongkai Zhu, Tianyu Feng, Zhengang Zhao, Jiafu Wei, Yong Peng, Yong Chen, Yuan Feng, Mao Chen

**Affiliations:** 1 Department of Cardiology, West China Hospital, Sichuan University, Chengdu 610041, China; zhuzhongkai@wchscu.cn (Z.Z.); zhaozhengang@foxmail.com (Z.Z.); weijiafu@163.com (J.W.); pengyongcd@126.com (Y.P.); 15320322354@163.com (Y.C.); fynotebook@hotmail.com (Y.F.); 2 Department of Laboratory Medicine, West China Hospital, Sichuan University, Chengdu 610041, China; tianyufeng@wchscu.cn; 3 Cardiac Structure and Function Research Key Laboratory of Sichuan Province, West China Hospital, Sichuan University, Chengdu 610041, China

**Keywords:** red cell distribution width, transcatheter aortic valve replacement, bicuspid aortic valve, longitudinal biomarker, mortality

## Abstract

**Background/Objectives**: Preprocedural red cell distribution width (RDW) is associated with mortality after transcatheter aortic valve replacement (TAVR), but the prognostic relevance of serial measurements and native leaflet morphology remains uncertain. **Methods**: This single-center retrospective cohort included 1369 patients who underwent native-valve TAVR from January 2019 to October 2023. The primary analysis evaluated baseline RDW-coefficient of variation (RDW-CV) and mortality in the overall cohort. Exploratory landmark analyses examined postprocedural RDW-CV changes among survivors with repeat measurements. Valve-disease phenotype and leaflet morphology were evaluated as potential effect modifiers. **Results**: During a median reverse Kaplan–Meier follow-up of 58.7 months, 196 patients died. Higher baseline RDW-CV was associated with mortality over all follow-up (adjusted HR per 1-SD increase, 1.17; 95% CI, 1.02–1.34; *p* = 0.022). After adjustment for baseline RDW-CV and clinical covariates, greater increases at 3 months (HR per window-specific 1-SD increase, 1.53; 95% CI, 1.20–1.94; *p* < 0.001) and 6 months (HR, 1.37; 95% CI, 1.08–1.73; *p* = 0.009) were associated with subsequent mortality. Exploratory analysis suggested a stronger 6-month association in bicuspid than tricuspid valves (HR, 2.47 [95% CI, 1.65–3.69] vs. 1.02 [0.75–1.40]; interaction *p* = 0.002). Adding 6-month RDW-CV change increased the cross-validated 2-year AUC from 0.628 to 0.651. **Conclusions**: Baseline RDW-CV was associated with mortality after TAVR. Among patients with repeat testing, increases at 3 and 6 months were associated with subsequent mortality, with an exploratory stronger association in bicuspid aortic valve. Standardized prospective sampling is required to confirm these longitudinal findings.

## 1. Introduction

Red cell distribution width (RDW) reflects variation in circulating erythrocyte size. Although routinely reported as part of the complete blood count, RDW is influenced by iron-restricted erythropoiesis, inflammation, renal dysfunction, nutritional status, and systemic congestion [1,2]. Higher preprocedural RDW has been associated with mortality after transcatheter aortic valve replacement (TAVR), suggesting that it captures aspects of physiological reserve not conveyed by hemoglobin alone [3,4].

Most TAVR studies have treated RDW as a preprocedural risk marker. Aung et al. extended this evidence by showing that a progressive postprocedural increase in RDW was associated with long-term mortality after TAVR [5]. Their study established the prognostic relevance of longitudinal RDW assessment, but did not determine whether its postprocedural association varies across anatomically distinct TAVR populations. This question is relevant because the biological and hemodynamic conditions represented by an elevated RDW-coefficient of variation (RDW-CV) may differ according to the anatomy in which the transcatheter prosthesis is implanted.

Native leaflet morphology provides an anatomical framework for examining this heterogeneity. Bicuspid and tricuspid valves differ in calcification pattern, annular geometry, associated aortopathy, and prosthesis–anatomy interaction [6,7,8,9]. These differences may influence post-TAVR gradients, paravalvular regurgitation, prosthesis expansion, and ventricular or vascular loading, thereby modifying the relationship between a systemic hematological marker and subsequent mortality. In contrast, classification as pure aortic stenosis (AS), mixed AS/aortic regurgitation (AR), or pure AR describes the dominant preprocedural hemodynamic lesion rather than leaflet architecture. Evaluating both classifications may therefore distinguish whether variation in the RDW-CV association is related primarily to the presenting valve lesion or to native anatomy interacting with the prosthesis.

The primary analysis evaluated the association between baseline RDW-CV and long-term mortality in the overall cohort. Among survivors with repeat measurements, exploratory landmark analyses examined whether postprocedural RDW-CV changes were associated with subsequent mortality after accounting for the baseline value. Native valve-disease phenotype and leaflet morphology were evaluated as potential effect modifiers. Readmission outcomes and incremental model performance were secondary analyses.

## 2. Materials and Methods

### 2.1. Study Design and Population

Consecutive patients who underwent TAVR for native aortic valve disease at West China Hospital, Sichuan University, from 1 January 2019 to 1 October 2023 were identified from the institutional TAVR database. Patients with baseline valve classification, preprocedural hematological assessment, and clinical follow-up were included in the analytic cohort. AS and AR severity were assigned from the preprocedural echocardiogram using guideline-recommended integrative criteria [10,11]. Pure AS denoted clinically relevant AS with no or trace AR, mixed AS/AR denoted coexisting clinically relevant AS and AR, and pure AR denoted clinically relevant AR without relevant AS. Native leaflet morphology was confirmed on preprocedural cardiac computed tomography and recorded as bicuspid (BAV), tricuspid (TAV), or quadricuspid [12]; quadricuspid cases were excluded only from BAV–TAV comparisons. Transcatheter prostheses comprised Venus, Prizvalve, VitaFlow, Evolut, Prostyle, SAPIEN 3, and Taurus devices, with nominal sizes of 20–34 mm. Device selection and sizing were determined by the treating Heart Team. The study was approved by the Ethics Committee of West China Hospital, Sichuan University [Approval No. 2024-2370; 12 December 2023], and informed consent was waived because of the retrospective design. Reporting follows STROBE [13].

### 2.2. RDW Measurements and Longitudinal Windows

All RDW-CV measurements were performed in the same institutional laboratory as part of routine clinical care. Preprocedural RDW-CV was analyzed as a continuous variable, standardized to 1 SD, and according to the previously reported threshold of 15.5% [3]. Postprocedural measurements were grouped as 0–7 days, 1 month (20–60 days), 3 months (75–120 days), 6 months (150–240 days), and 12 months (300–450 days). The longitudinal dataset contained one RDW-CV result per available window; landmark analyses required a valid sampling day. For each landmark analysis, eligible patients had survived to the sampling date and had both a baseline RDW-CV value and a valid dated measurement within the corresponding window. RDW-CV change was calculated as the absolute difference from baseline in percentage points and standardized separately using the SD of change within each landmark cohort. The sampling date defined time zero, and only subsequent deaths contributed to the landmark analysis.

### 2.3. Outcomes

The primary outcome was all-cause mortality, ascertained from the adjudicated death status and date recorded in the institutional TAVR database. Associations were estimated over available follow-up and at 1, 2, 3, and 5 years. Administrative follow-up ended on 31 July 2026; patients without a recorded death were censored on that date. Potential follow-up was summarized using the reverse Kaplan–Meier method, and numbers remaining under observation were reported at the prespecified mortality horizons. Secondary outcomes were assessed at 1 year and included unplanned emergency-department or inpatient readmission, cardiovascular readmission, and the corresponding time-to-first-event composites with death. Definitions followed contemporary TAVR standards where applicable [14].

### 2.4. Statistical Analysis

Continuous variables are reported as medians with interquartile ranges and categorical variables as counts with percentages. Cox models estimated hazard ratios and 95% confidence intervals [15]. Covariates were selected a priori according to clinical relevance and availability. Models included age, sex, valve-disease phenotype, estimated glomerular filtration rate, hemoglobin, and mean corpuscular volume; change models additionally included baseline RDW-CV. Model complexity was restricted in landmark and subgroup analyses with fewer deaths. Proportional-hazards assumptions were assessed using Schoenfeld residuals. Effect modification was evaluated in pooled models using interaction terms and a common exposure scale. Primary models used complete cases without covariate imputation. Measurement availability and inverse-probability weighting were assessed among survivors at each window’s end (days 120, 240, and 450), separately from the sampling-date landmark analyses. Additional sensitivity analyses included a time-updated Cox model using the most recent observed RDW-CV and a joint longitudinal–survival model evaluating the model-estimated current RDW-CV level and linear slope [16]. Predictive performance was evaluated separately using patient-level repeated five-fold cross-validation of the C-index and 2-year time-dependent area under the curve. No multiplicity adjustment was applied to secondary endpoint and interaction analyses, and these findings were interpreted as exploratory. Analyses were performed using R version 4.4.2. All tests were two-sided.

## 3. Results

### 3.1. Cohort and Baseline RDW

**Figure 1:**
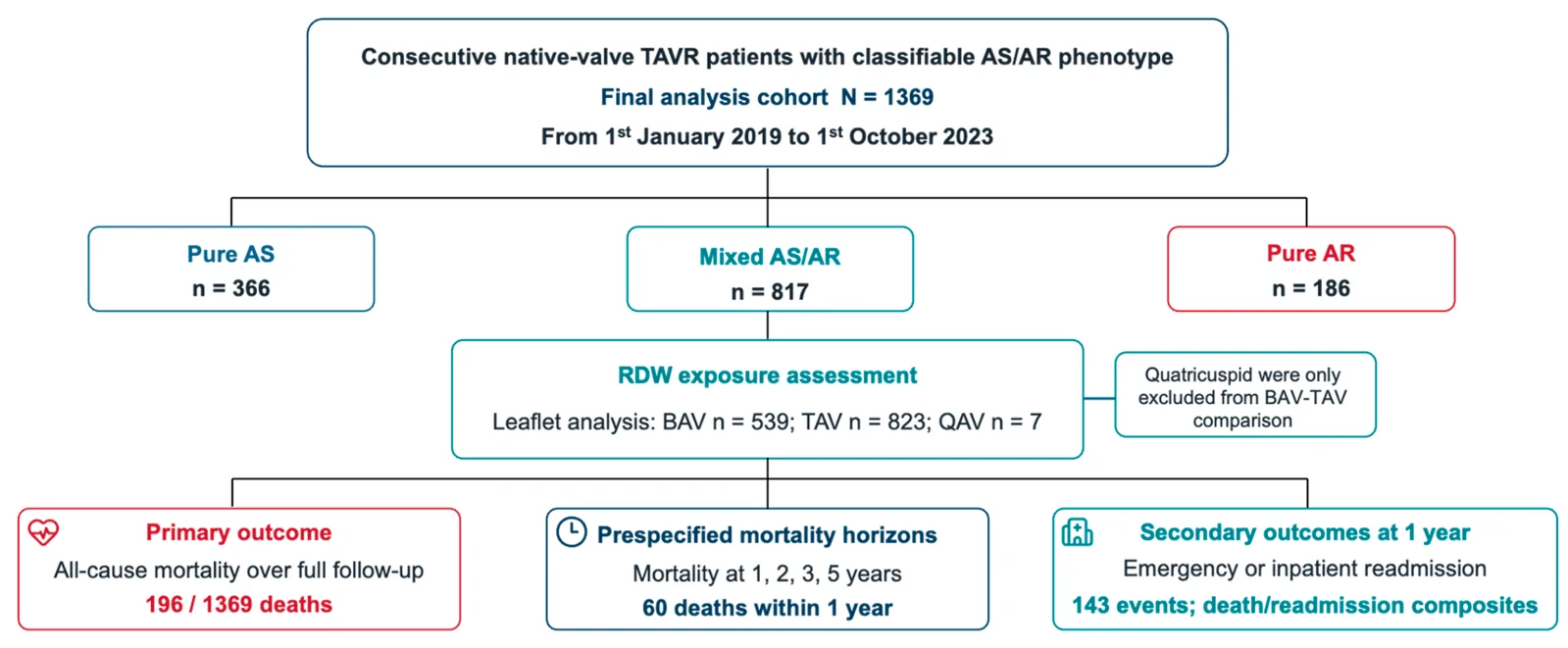
Study cohort, native valve-disease phenotypes, leaflet morphology, and study outcomes. The cohort comprised 1369 patients; screening and exclusion records were unavailable. AS, aortic stenosis; AR, aortic regurgitation; BAV, bicuspid aortic valve; TAV, tricuspid aortic valve; QAV, quadricuspid aortic valve; RDW, red cell distribution width.

The analytic cohort included 1369 patients: 366 with pure AS, 817 with mixed AS/AR, and 186 with pure AR (Figure 1). Leaflet morphology was tricuspid in 823 patients, bicuspid in 539, and quadricuspid in 7. The seven patients with quadricuspid valves were excluded only from the BAV–TAV analyses. Procedural characteristics are summarized in Table S1. Follow-up ended on 31 July 2026; median follow-up by the reverse Kaplan–Meier method was 58.7 months. During follow-up, 196 deaths were recorded, including 60 within 1 year. Baseline characteristics according to the 15.5% RDW-CV threshold are shown in Table 1 and Table S6A.

### 3.2. Baseline RDW, Mortality and Valve-Disease Phenotype

**Figure 2:**
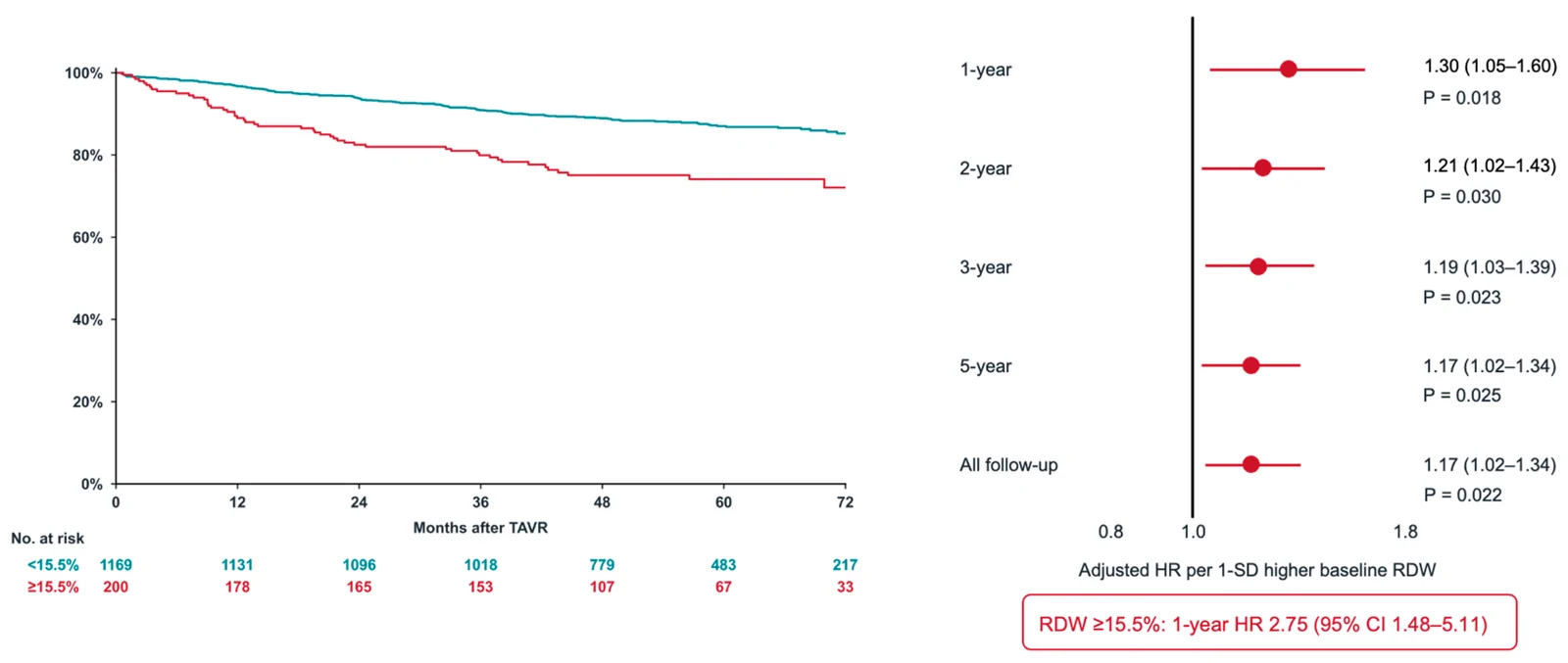
Baseline RDW-CV and mortality. Kaplan–Meier survival according to the preprocedural RDW-CV threshold and adjusted mortality associations per 1-SD higher baseline RDW-CV at 1, 2, 3, and 5 years and over all follow-up. The SD of baseline RDW-CV was 1.90 percentage points. CI, confidence interval; HR, hazard ratio; RDW-CV, red cell distribution width coefficient of variation; SD, standard deviation.

Higher baseline RDW-CV was associated with mortality over all available follow-up and at the prespecified time horizons. The adjusted HR per 1-SD increase was 1.30 (95% CI, 1.05–1.60; *p* = 0.018) at 1 year, 1.21 (95% CI, 1.02–1.43; *p* = 0.030) at 2 years, 1.19 (95% CI, 1.03–1.39; *p* = 0.023) at 3 years, 1.17 (95% CI, 1.02–1.34; *p* = 0.025) at 5 years, and 1.17 (95% CI, 1.02–1.34; *p* = 0.022) over all follow-up. Patients with RDW-CV ≥15.5% had lower observed survival and higher 1-year mortality (adjusted HR, 2.75; 95% CI, 1.48–5.11; *p* = 0.001) (Figure 2). Phenotype-specific estimates were largest in pure AR, although interaction tests were not significant at 1 year, 5 years, or over all follow-up (Figure S1 and Table S2). Sequential models are reported in Table S3.

### 3.3. Associations of RDW-CV Change with Subsequent Mortality Across Measurement Windows

**Figure 3:**
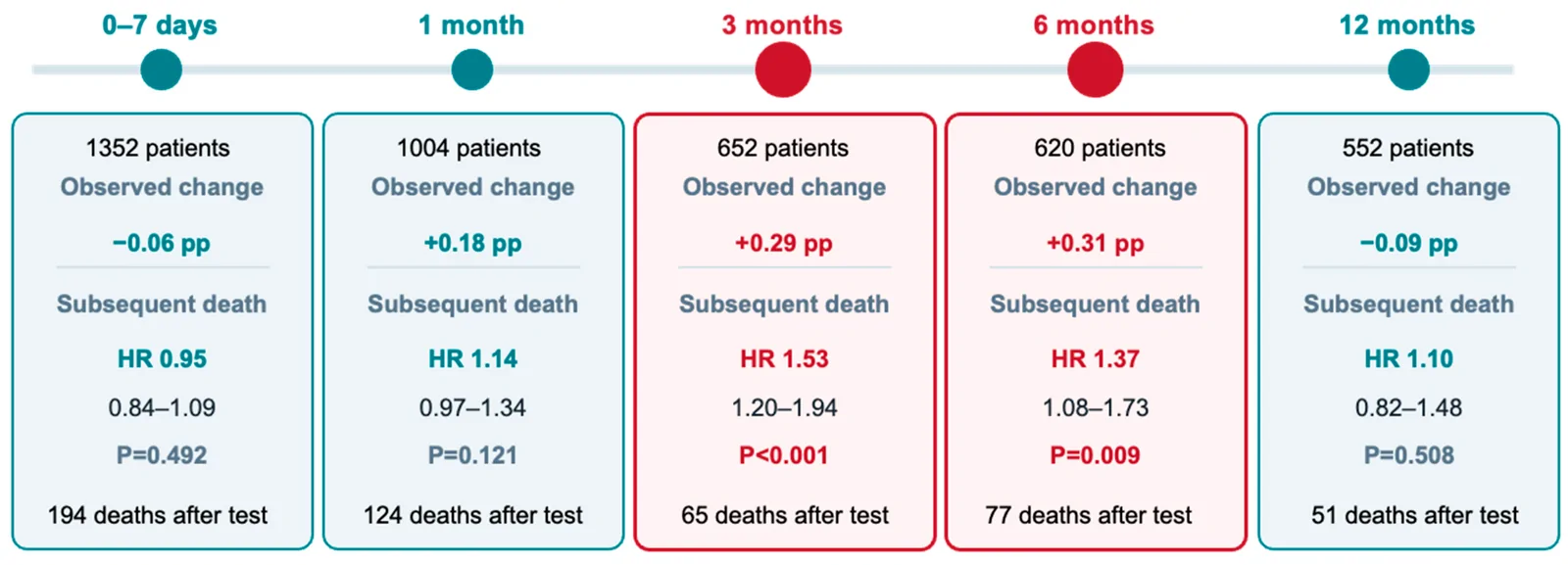
Adjusted associations between RDW-CV change and subsequent mortality across landmark windows. Estimates derive from separate landmark cohorts and are expressed per window-specific 1-SD increase in RDW-CV change. The SDs of RDW-CV change were 0.63, 1.20, 1.41, 1.66, and 1.50 percentage points for the 0–7-day, 1-, 3-, 6-, and 12-month windows, respectively. Three quadricuspid cases were excluded from the 6-month BAV–TAV comparison (620 to 617 patients). pp, percentage points. CI, confidence interval; HR, hazard ratio; RDW-CV, red cell distribution width coefficient of variation; TAVR, transcatheter aortic valve replacement.

Landmark analyses were restricted to survivors with an available repeat RDW-CV measurement in the corresponding window. The adjusted HRs per window-specific 1-SD increase were 0.95 (95% CI, 0.84–1.09) at 0–7 days, 1.14 (95% CI, 0.97–1.34) at 1 month, 1.53 (95% CI, 1.20–1.94) at 3 months, 1.37 (95% CI, 1.08–1.73) at 6 months, and 1.10 (95% CI, 0.82–1.48) at 12 months (Figure 3). Because each window comprised a separately assembled landmark cohort, these estimates represent window-specific associations rather than direct comparisons of effect across time. Full estimates are reported in Table S4.

### 3.4. Exploratory Six-Month Association in BAV and TAV

**Figure 4:**
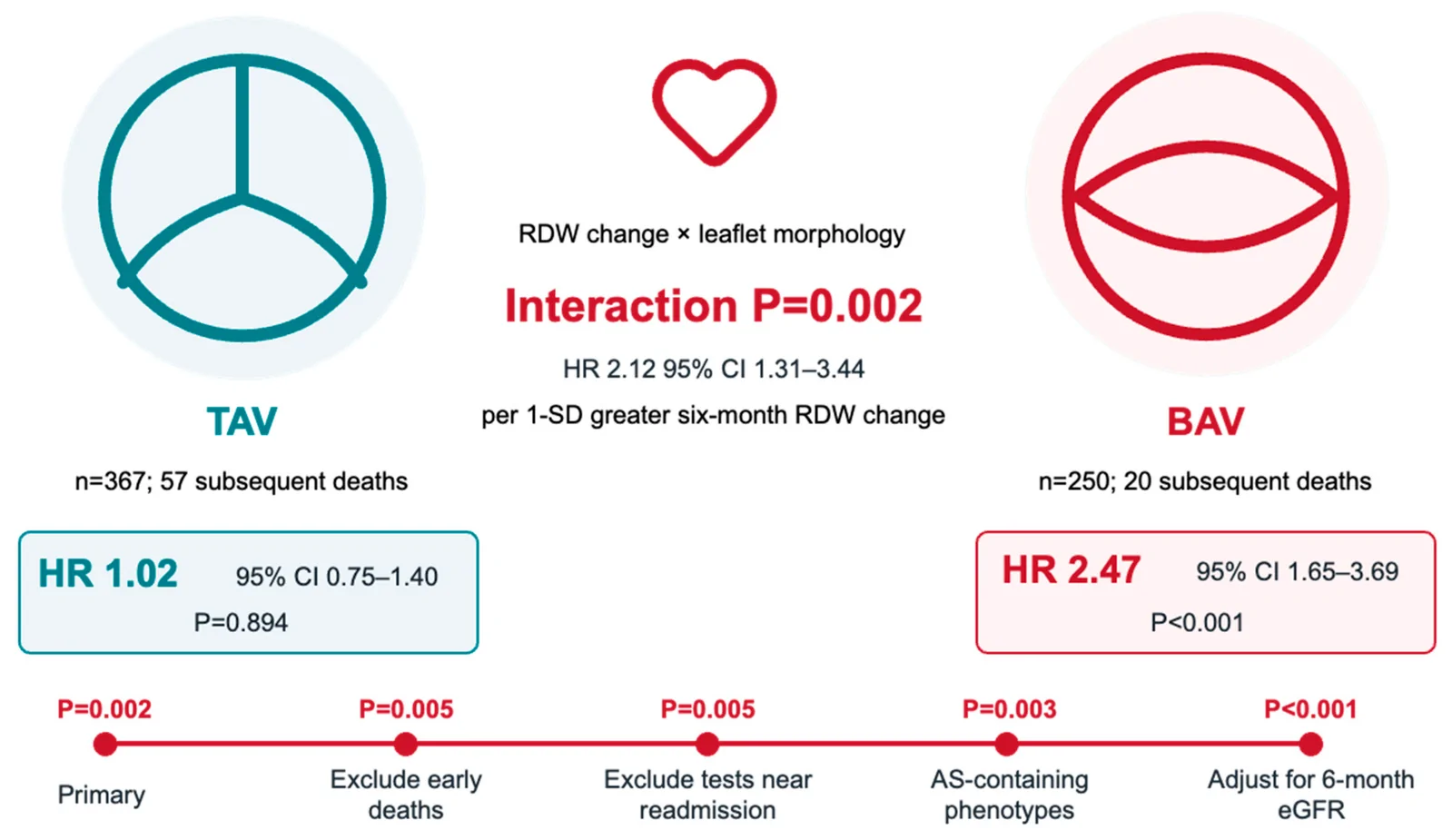
Exploratory interaction between 6-month RDW-CV change and leaflet morphology. Morphology-specific estimates used the same 1-SD increment. The sensitivity estimates are not independent replications. BAV, bicuspid aortic valve; HR, hazard ratio; RDW-CV, red cell distribution width coefficient of variation; TAV, tricuspid aortic valve.

In a pooled interaction model, leaflet morphology modified the association between 6-month RDW-CV change and subsequent mortality (interaction HR, 2.12; 95% CI, 1.31–3.44; *p* = 0.002) (Figure 4). On the common 1-SD scale, the corresponding HRs were 2.47 (95% CI, 1.65–3.69; *p* < 0.001) in BAV and 1.02 (95% CI, 0.75–1.40; *p* = 0.894) in TAV. Prespecified sensitivity analyses produced similar interaction estimates (Table S2). The BAV estimate was based on 20 subsequent deaths. Additional adjustment for eGFR, NLR, and platelet count produced a similar interaction estimate (HR, 2.33; 95% CI, 1.42–3.81; *p* < 0.001), as did the other sensitivity analyses reported in Table S2. The interaction remained evident after additional adjustment for prosthesis expansion mechanism and STS-PROM (interaction HR, 2.10; 95% CI, 1.30–3.40; *p* = 0.002; Table S6B). Given the limited number of BAV events, incomplete procedural data, and absence of external validation, this finding remains exploratory.

### 3.5. Longitudinal RDW-CV Measures and Incremental Performance

**Figure 5:**
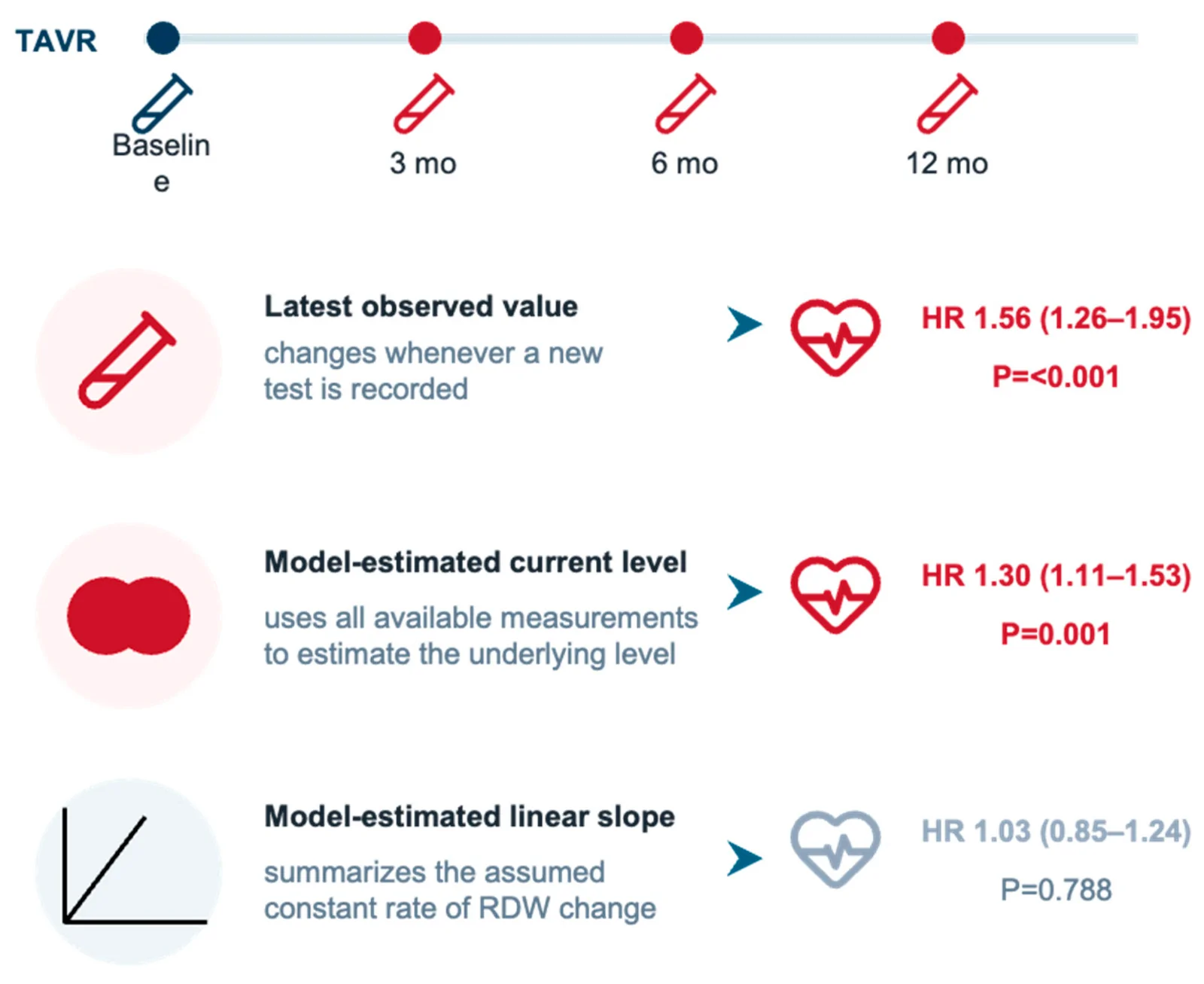
Longitudinal RDW-CV exposure models and subsequent mortality. Adjusted hazard ratios for subsequent mortality according to the latest observed RDW-CV, model-estimated current RDW-CV level, and model-estimated linear slope. CI, confidence interval; HR, hazard ratio; RDW-CV, red cell distribution width coefficient of variation.

The most recent observed RDW-CV value remained associated with mortality after baseline adjustment (HR, 1.56; 95% CI, 1.26–1.95; *p* < 0.001). In the joint longitudinal–survival model, the model-estimated current level was associated with mortality (HR, 1.30; 95% CI, 1.11–1.53; *p* = 0.001), whereas the estimated linear slope was imprecise (HR, 1.03; 95% CI, 0.85–1.24; *p* = 0.788) (Figure 5).

Follow-up RDW-CV was available in approximately half of survivors at the ends of the 3-, 6-, and 12-month windows (Figure S2). Missing sampling dates and deaths before window closure account for differences from the sampling-date landmark cohorts. Inverse-probability weighting yielded similar associations at 3 and 6 months (Table S4). Adding 6-month RDW-CV change produced a modest gain in cross-validated discrimination (ΔAUC at 2 years, 0.024; 95% CI, −0.019 to 0.069; Table S5).

### 3.6. One-Year Mortality and Readmission Outcomes

At 1 year, baseline RDW-CV was associated with all-cause mortality. No clear association was detected for emergency or inpatient readmission, cardiovascular readmission, or either composite outcome (Table 2). The confidence intervals remained compatible with modest associations.

## 4. Discussion

Baseline RDW-CV was associated with mortality after TAVR, while increases at 3 and 6 months were associated with subsequent mortality among survivors with repeat testing. The most recent and model-estimated current levels carried the longitudinal signal. The stronger 6-month association in BAV was hypothesis-generating. The main findings are summarized in the Graphical Abstract.

Previous studies established baseline RDW as a mortality marker after TAVR [3,4], and Aung et al. associated a progressive postprocedural rise with adverse outcome [5]. The present study extends that work by examining defined postprocedural windows and by separating the current RDW-CV level from an assumed constant trajectory. The separate landmark estimates do not establish that the association began at 3 months or disappeared at 12 months, because the tested cohorts differed and the confidence intervals overlapped. They show that RDW-CV changes measured at 3 and 6 months retained prognostic information after adjustment for baseline RDW-CV.

Early RDW-CV measurements may reflect blood loss, transfusion, fluid shifts, acute kidney injury, and procedural inflammation, whereas later values may reflect persistent disturbances in erythropoiesis and systemic health. Subclinical hemolysis after TAVR may also contribute to erythrocyte abnormalities [17]. High shear stress in aortic valve disease may cause loss of high-molecular-weight von Willebrand factor multimers and predispose susceptible patients to gastrointestinal bleeding, as recognized in Heyde syndrome [18]. Recurrent or occult blood loss may lead to iron-restricted erythropoiesis and greater variation in erythrocyte size, providing a plausible link between gastrointestinal bleeding, iron deficiency, and elevated RDW-CV. Although TAVR may correct the hemostatic abnormality, persistent or recurrent bleeding could contribute to an elevated recovery-phase RDW-CV; this mechanism could not be examined directly because von Willebrand factor, iron indices, and bleeding severity were not systematically available. A prospective intensive-care study similarly associated elevated RDW with inflammatory and metabolic abnormalities and mortality, supporting its broader interpretation as a marker of systemic vulnerability [19], although that population differs substantially from patients undergoing TAVR. RDW-CV should therefore be interpreted as an integrative marker rather than evidence of a specific biological mechanism. The association with the current RDW-CV level, but not a constant linear slope, is also consistent with a nonlinear and irregularly sampled postprocedural course. Similar prognostic patterns have been reported for serial natriuretic peptide measurements after TAVR [20,21].

Leaflet morphology may provide an anatomical context for the recovery-phase association between RDW-CV and mortality. Bicuspid and tricuspid valves differ in calcification pattern, annular geometry, aortopathy, and prosthesis–anatomy interaction [6,7,8,9,22,23], which may influence the hemodynamic conditions under which systemic recovery occurs after TAVR. In this study, the association between 6-month RDW-CV change and subsequent mortality was stronger in BAV than in TAV and remained evident after adjustment for concurrent eGFR, NLR, and platelet count. However, this interaction was confined to one landmark window and was based on 20 subsequent deaths in the BAV group. It should therefore be regarded as a hypothesis-generating observation that supports prospective studies combining scheduled RDW-CV measurements with post-TAVR imaging and hemodynamic assessment, rather than as evidence that BAV independently increases mortality or requires a morphology-specific RDW-CV threshold.

Native valve-disease phenotype and leaflet morphology captured different dimensions of risk. Baseline associations were numerically largest in pure AR, but interaction tests did not support differences across pure AS, mixed AS/AR, and pure AR. In contrast, the exploratory 6-month association differed by leaflet morphology. Thus, the preprocedural hemodynamic lesion and the anatomy interacting with the prosthesis are not interchangeable risk constructs. The pure-AR estimate remains descriptive rather than threshold-defining. Dedicated AR devices were used in only eight patients, limiting device-specific comparisons.

The potential clinical value of serial RDW-CV lies in complementing established assessment rather than providing stand-alone prediction. Baseline RDW-CV was not clearly associated with readmission, although the confidence intervals remained compatible with modest effects; these results do not establish endpoint specificity. The cross-validated improvement from 6-month change was modest. Prospective studies are needed to determine whether standardized serial RDW-CV measurements provide clinically useful information beyond routine clinical and imaging assessment.

The single-center design, limited BAV events, multiple secondary comparisons, and internal-only validation limit generalizability. Follow-up RDW-CV was available in only approximately half of eligible survivors, potentially introducing informative selection; landmark findings therefore apply only to survivors with available measurements and cannot be generalized to all TAVR patients. Due to incomplete data regarding hemolysis markers, iron metabolism parameters, and serial imaging, the aforementioned mechanisms could not be directly evaluated. Therefore, the concepts of morphological interaction and isolated AR patterns should be regarded as hypotheses requiring prospective validation. The modest discrimination gains also limit conclusions about clinical utility. A study incorporating scheduled blood sampling and concurrent echocardiography at 3 and 6 months would help clarify whether RDW-CV provides reproducible information beyond established clinical and imaging assessments, and whether correcting potential abnormalities alters risk. BAV subtypes could not be determined in some patients, and calcification was quantified by total volume without assessment of its spatial distribution. EuroSCORE and standardized frailty scores were not consistently available, and device-specific pure-AR comparisons could not be assessed reliably.

## 5. Conclusions

Higher baseline RDW-CV was associated with mortality after TAVR. Increases at 3 and 6 months were associated with subsequent mortality, although these landmark findings apply only to survivors with available measurements. The stronger 6-month association in BAV than TAV remains hypothesis-generating given the sparse BAV events. Discrimination gains were modest. Prospective studies with standardized sampling and external validation are needed.

## Figures and Tables

**Table 1:** Baseline characteristics according to preprocedural RDW-CV.

Characteristic	Overall (*N* = 1369)	RDW < 15.5% (*N* = 1169)	RDW ≥ 15.5% (*N* = 200)
Age, years	73.0 (68.0–78.0)	73.0 (68.0–78.0)	74.0 (68.0–80.0)
Male sex	813 (59.4)	698 (59.7)	115 (57.5)
Body mass index, kg/m^2^	22.7 (20.3–25.0)	22.9 (20.5–25.3)	22.0 (19.1–23.9)
Pure AS	366 (26.7)	316 (27.0)	50 (25.0)
Mixed AS/AR	817 (59.7)	698 (59.7)	119 (59.5)
Pure AR	186 (13.6)	155 (13.3)	31 (15.5)
Diabetes mellitus	235 (17.2)	205 (17.5)	30 (15.0)
Hypertension	609 (44.6)	527 (45.2)	82 (41.0)
Coronary artery disease	295 (21.5)	253 (21.6)	42 (21.0)
Atrial fibrillation	208 (15.2)	163 (13.9)	45 (22.6)
STS-PROM, %	2.9 (1.9–4.9)	2.8 (1.8–4.7)	3.7 (2.4–6.4)
LVEF, %	60.0 (44.0–67.0)	60.0 (45.0–67.0)	55.0 (38.0–65.2)
eGFR, mL/min/1.73 m^2^	70.7 (57.6–84.5)	71.3 (59.3–84.5)	65.3 (45.9–82.0)
Hemoglobin, g/L	130.0 (117.0–141.0)	131.0 (120.0–142.0)	110.5 (92.0–129.2)
MCV, fL	93.5 (90.1–96.8)	93.8 (90.8–97.0)	89.3 (78.4–95.7)
RDW-CV, %	13.6 (13.0–14.6)	13.4 (12.8–14.2)	16.6 (15.9–18.3)
Albumin, g/L	41.8 (39.1–44.4)	42.3 (39.5–44.7)	39.8 (36.3–42.6)
NLR	2.6 (1.9–3.7)	2.6 (1.9–3.6)	2.9 (2.1–5.4)

Values are median (interquartile range) or *n* (%). Variable-specific denominators were: body mass index, 1367 overall/1168 RDW-CV < 15.5%/199 RDW-CV ≥ 15.5%; hypertension, 1367/1167/200; atrial fibrillation, 1368/1169/199; and NLR, 1357/1164/193. AS, aortic stenosis; AR, aortic regurgitation; eGFR, estimated glomerular filtration rate; LVEF, left ventricular ejection fraction; MCV, mean corpuscular volume; NLR, neutrophil-to-lymphocyte ratio; RDW-CV, red cell distribution width coefficient of variation; STS-PROM, Society of Thoracic Surgeons Predicted Risk of Mortality.

**Table 2:** One-year associations of baseline RDW-CV with mortality, readmission, and composite outcomes.

Endpoint	Exposure	*N*	Events	Adjusted HR (95% CI)	*p* Value
All-cause death	Per 1-SD RDW-CV	1369	60	1.30 (1.05–1.60)	0.018
All-cause death	RDW-CV ≥ 15.5%	1369	60	2.75 (1.48–5.11)	0.001
Any ED/inpatient readmission	Per 1-SD RDW-CV	1369	143	0.93 (0.76–1.14)	0.506
Any ED/inpatient readmission	RDW-CV ≥ 15.5%	1369	143	0.77 (0.45–1.32)	0.340
Death or any ED/inpatient readmission	Per 1-SD RDW-CV	1369	188	1.08 (0.93–1.26)	0.299
Death or any ED/inpatient readmission	RDW-CV ≥ 15.5%	1369	188	1.25 (0.83–1.87)	0.294
Strict cardiovascular readmission	Per 1-SD RDW-CV	1369	60	0.96 (0.70–1.30)	0.778
Strict cardiovascular readmission	RDW-CV ≥ 15.5%	1369	60	0.50 (0.20–1.28)	0.150
Death or strict cardiovascular readmission	Per 1-SD RDW-CV	1369	112	1.17 (0.98–1.40)	0.077
Death or strict cardiovascular readmission	RDW-CV ≥ 15.5%	1369	112	1.49 (0.90–2.46)	0.123

Estimates are restricted to 1-year outcomes. Models adjust for age, sex, valve phenotype, estimated glomerular filtration rate, hemoglobin, and mean corpuscular volume. Counts represent patients with at least one event; composites were analyzed as time to first event. In readmission-only models, death before readmission was treated as censoring. ED/inpatient readmission excludes outpatient visits and the index TAVR admission. CI, confidence interval; ED, emergency department; HR, hazard ratio; RDW-CV, red cell distribution width coefficient of variation; SD, standard deviation.

## Data Availability

The data presented in this study are available from the corresponding author on reasonable request.

## Supplementary Materials

The following supporting information can be downloaded at: https://www.mdpi.com/article/10.3390/diagnostics16213659/s1, Figure S1, Phenotype-specific associations between baseline RDW-CV and mortality; Figure S2, Availability of RDW-CV measurements at 3, 6, and 12 months and standardized differences between patients with and without measurements; Table S1, TAVR procedural characteristics; Table S2, Effect-modification analyses by native valve-disease phenotype and leaflet morphology; Table S3, Sequential baseline RDW-CV models; Table S4, General landmark sensitivity analyses; Table S5, Incremental predictive performance of recovery-phase RDW-CV change; Table S6, Valve anatomy, prosthesis use, surgical risk, and adjusted sensitivity analyses.

## Abbreviations

The following abbreviations are used in this manuscript:

AR	aortic regurgitation
AS	aortic stenosis
BAV	bicuspid aortic valve
CI	confidence interval
eGFR	estimated glomerular filtration rate
HR	hazard ratio
MCV	mean corpuscular volume
RDW	red cell distribution width
TAV	tricuspid aortic valve
TAVR	transcatheter aortic valve replacement
