# Supplementary material for: Serial Red Cell Distribution Width and Mortality After TAVR: An Observational Cohort Study
Source: Diagnostics (Basel). 2026 Nov 5;16(21):3659. doi: 10.3390/diagnostics16213659 (PMC13763372; doi:10.3390/diagnostics16213659)
Supplement: Supplementary file 1 [file diagnostics-16-03659-s001.zip › diagnostics-4630939-supplementary.pdf]

Serial Red Cell Distribution Width and Mortality after TAVR: An Observational Cohort Study

Table S1. TAVR procedural characteristics

Characteristic	Value	Characteristic	Value
THV brand		Annular rupture	2 (0.1)
Venus	943 (69)	Cardiac tamponade	4 (0.3)
Taurus	121 (8.9)	Vascular complication	
Prizvalve	106 (7.7)	Minor	51 (3.7)
VitaFlow	94 (6.9)	Major	11 (0.8)
Evolut	62 (4.5)	Bleeding event	
ProStyle	30 (2.2)	Minor	24 (1.8)
SAPIEN 3	11 (0.8)	Major	7 (0.5)
Nominal valve size, mm	range, 20–34	Coronary obstruction	23 (1.7)
Coronary protection	212 (15.5)	New PPMI	196 (14.3)
Protected coronary artery	185 (13.5)	Any stroke	11 (0.8)
THV number > 1	208 (15.6)	In-hospital death	15 (1.1)
Conversion to surgery	4 (0.3)		

Data are n (%) or median (interquartile range). PPMI: permanent pacemaker implantation; THV: transcatheter heart valve.

Table S2. Effect-modification analyses by native valve-disease phenotype and leaflet morphology.

Domain	Analysis	Endpoint/window	Estimate	N	Events	HR (95% CI)	P value
AS/AR phenotype	Baseline RDW	1-year mortality	Interaction	1369	60	—	0.465
AS/AR phenotype	Baseline RDW	5-year mortality	Interaction	1369	188	—	0.284
AS/AR phenotype	Baseline RDW	All follow-up mortality	Interaction	1369	196	—	0.230
AS/AR phenotype	RDW change	3-month landmark	Interaction	652	65	—	0.078
AS/AR phenotype	RDW change	6-month landmark	Interaction	620	77	—	0.472
Leaflet morphology	Primary	6-month landmark	Interaction	617	77	—	0.002
Leaflet morphology	Primary	6-month landmark	TAV	367	57	1.02 (0.75–1.40)	0.894
Leaflet morphology	Primary	6-month landmark	BAV	250	20	2.47 (1.65–3.69)	<0.001
Leaflet morphology	Exclude death within 30 days	6-month landmark	Interaction	615	75	—	0.005
Leaflet morphology	Exclude death within 30 days	6-month landmark	TAV	366	56	0.93 (0.67–1.29)	0.669
Leaflet morphology	Exclude death within 30 days	6-month landmark	BAV	249	19	2.41 (1.56–3.71)	<0.001
Leaflet morphology	Exclude RDW within 14 days of readmission	6-month landmark	Interaction	601	71	—	0.005
Leaflet morphology	Exclude RDW within 14 days of readmission	6-month landmark	TAV	354	51	1.12 (0.81–1.54)	0.500
Leaflet morphology	Exclude RDW within 14 days of readmission	6-month landmark	BAV	247	20	2.44 (1.63–3.64)	<0.001
Leaflet morphology	Restrict to AS-containing phenotypes	6-month landmark	Interaction	529	64	—	0.003
Leaflet morphology	Restrict to AS-containing phenotypes	6-month landmark	TAV	282	44	0.99 (0.69–1.41)	0.948
Leaflet morphology	Restrict to AS-containing phenotypes	6-month landmark	BAV	247	20	2.54 (1.68–3.85)	<0.001
Leaflet morphology	Additional 6-month eGFR adjustment	6-month landmark	Interaction	611	75	—	<0.001
Leaflet morphology	Additional 6-month eGFR adjustment	6-month landmark	TAV	362	55	0.92 (0.66–1.27)	0.605
Leaflet morphology	Additional 6-month eGFR adjustment	6-month landmark	BAV	249	20	2.24 (1.49–3.35)	<0.001
Leaflet morphology	Concurrent 6-month eGFR, NLR, and platelet adjustment	6-month landmark	Interaction	607	73	2.33 (1.42-3.81)	<0.001

Interaction rows report product-term P values. Morphology-specific hazard ratios are shown per 1-standard-deviation increase in RDW-CV change. The final interaction estimate is the exponentiated product term after simultaneous adjustment for concurrent eGFR, NLR, and platelet count. AS, aortic stenosis; AR, aortic regurgitation; BAV, bicuspid aortic valve; NLR, neutrophil-to-lymphocyte ratio; TAV, tricuspid aortic valve.

Table S3. Sequential baseline RDW-CV models.

Endpoint	Exposure	Model	N	Events	HR (95% CI)	P value
1-year mortality	Per 1-SD RDW	M1	1369	60	1.36 (1.17–1.59)	<0.001
1-year mortality	Per 1-SD RDW	M2	1369	60	1.30 (1.09–1.55)	0.003
1-year mortality	Per 1-SD RDW	M3	1369	60	1.30 (1.05–1.60)	0.018
1-year mortality	RDW-CV ≥15.5%	M1	1369	60	3.29 (1.94–5.59)	<0.001
1-year mortality	RDW-CV ≥15.5%	M2	1369	60	2.63 (1.52–4.55)	<0.001
1-year mortality	RDW-CV ≥15.5%	M3	1369	60	2.75 (1.48–5.11)	0.001
2-year mortality	Per 1-SD RDW	M1	1369	108	1.31 (1.15–1.49)	<0.001
2-year mortality	Per 1-SD RDW	M2	1369	108	1.27 (1.11–1.45)	<0.001
2-year mortality	Per 1-SD RDW	M3	1369	108	1.21 (1.02–1.43)	0.030
2-year mortality	RDW-CV ≥15.5%	M1	1369	108	2.81 (1.87–4.21)	<0.001
2-year mortality	RDW-CV ≥15.5%	M2	1369	108	2.45 (1.62–3.71)	<0.001
2-year mortality	RDW-CV ≥15.5%	M3	1369	108	2.36 (1.46–3.81)	<0.001
3-year mortality	Per 1-SD RDW	M1	1369	146	1.31 (1.17–1.46)	<0.001
3-year mortality	Per 1-SD RDW	M2	1369	146	1.26 (1.12–1.42)	<0.001
3-year mortality	Per 1-SD RDW	M3	1369	146	1.19 (1.02–1.39)	0.023
3-year mortality	RDW-CV ≥15.5%	M1	1369	146	2.25 (1.56–3.24)	<0.001
3-year mortality	RDW-CV ≥15.5%	M2	1369	146	1.93 (1.33–2.80)	<0.001
3-year mortality	RDW-CV ≥15.5%	M3	1369	146	1.69 (1.09–2.62)	0.018
5-year mortality	Per 1-SD RDW	M1	1369	188	1.29 (1.17–1.43)	<0.001
5-year mortality	Per 1-SD RDW	M2	1369	188	1.25 (1.13–1.40)	<0.001
5-year mortality	Per 1-SD RDW	M3	1369	188	1.17 (1.02–1.34)	0.025
5-year mortality	RDW-CV ≥15.5%	M1	1369	188	2.13 (1.53–2.95)	<0.001
5-year mortality	RDW-CV ≥15.5%	M2	1369	188	1.89 (1.35–2.64)	<0.001
5-year mortality	RDW-CV ≥15.5%	M3	1369	188	1.59 (1.07–2.35)	0.021
All follow-up mortality	Per 1-SD RDW	M1	1369	196	1.28 (1.16–1.42)	<0.001
All follow-up mortality	Per 1-SD RDW	M2	1369	196	1.25 (1.12–1.39)	<0.001
All follow-up mortality	Per 1-SD RDW	M3	1369	196	1.17 (1.02–1.34)	0.022
All follow-up mortality	RDW-CV ≥15.5%	M1	1369	196	2.08 (1.51–2.88)	<0.001
All follow-up mortality	RDW-CV ≥15.5%	M2	1369	196	1.87 (1.34–2.59)	<0.001
All follow-up mortality	RDW-CV ≥15.5%	M3	1369	196	1.58 (1.08–2.33)	0.019

M1 includes age and sex; M2 adds AS/AR phenotype; M3 adds eGFR, haemoglobin, and MCV.

Table S4. General landmark sensitivity analyses.

Window	Sensitivity analysis	N	Events	HR (95% CI)	P value
0–7 days	Exclude measurement within ±14 d of first readmission	1329	191	0.95 (0.84–1.09)	0.486
0–7 days	Exclude deaths within 30 d after measurement	1340	182	0.96 (0.84–1.10)	0.526
1 month	Exclude measurement within ±14 d of first readmission	964	112	1.12 (0.94–1.33)	0.190
1 month	Exclude deaths within 30 d after measurement	999	120	1.15 (0.97–1.36)	0.103
3 months	Exclude measurement within ±14 d of first readmission	638	63	1.50 (1.18–1.91)	0.001
3 months	Exclude deaths within 30 d after measurement	649	62	1.45 (1.13–1.86)	0.004
6 months	Exclude measurement within ±14 d of first readmission	604	71	1.46 (1.15–1.85)	0.002
6 months	Exclude deaths within 30 d after measurement	618	75	1.26 (0.98–1.62)	0.073
12 months	Exclude measurement within ±14 d of first readmission	540	49	1.07 (0.78–1.45)	0.683
12 months	Exclude deaths within 30 d after measurement	551	50	1.12 (0.83–1.51)	0.464
3 months	IPW for follow-up RDW availability	651	64	1.56 (1.20–2.03)	<0.001
6 months	IPW for follow-up RDW availability	617	74	1.30 (1.02–1.65)	0.032

IPW, inverse-probability weighting.

Table S5. Incremental predictive performance of recovery-phase RDW-CV change.

Window	Model	N / deaths / 2-y deaths	C-index	LR P	CV 2-y AUC	ΔAUC (95% CI)	CV Brier	ΔBrier (95% CI)
3 months	Clinical M3	652 / 65 / 35	0.665	—	0.559	Reference	0.0506	Reference
3 months	M3 + baseline RDW	652 / 65 / 35	0.670	0.450	0.553	Reference	0.0507	Reference
3 months	M3 + baseline RDW + RDW change	652 / 65 / 35	0.693	<0.001	0.576	0.023 (−0.038 to 0.079)	0.0494	−0.0013 (−0.0033 to 0.0005)
6 months	Clinical M3	620 / 77 / 36	0.670	—	0.631	Reference	0.0533	Reference
6 months	M3 + baseline RDW	620 / 77 / 36	0.676	0.572	0.628	Reference	0.0535	Reference
6 months	M3 + baseline RDW + RDW change	620 / 77 / 36	0.701	0.015	0.651	0.024 (−0.019 to 0.069)	0.0532	−0.0003 (−0.0026 to 0.0017)

Likelihood-ratio P values compare each model with the preceding model. Cross-validated changes are relative to the model containing clinical covariates and baseline RDW-CV. M3 includes age, sex, valve phenotype, eGFR, haemoglobin, and MCV. CI, confidence interval; CV, cross-validated.

Table S6. Valve anatomy, prosthesis use, surgical risk, and adjusted sensitivity analyses.

A. Pure AS and pure AR characteristics

Characteristic	Pure AS (n=366)	Pure AR (n=186)	P value
Leaflet morphology			<0.001
BAV	264 (72.1)	8 (4.3)	—
TAV	101 (27.6)	174 (93.5)	—
Quadricuspid	1 (0.3)	4 (2.2)	—
Unicuspid	0	0	—
BAV subtype			0.006
Type 0	168 (63.6)	1 (12.5)	-
Type 1	74 (28.0)	7 (87.5)	-
Type 2	10 (3.8)	0	-
Functional BAV	6 (2.3)	0	-
Unclassified	6 (2.3)	0	-
Calcification volume, mm^3	300.90 (171.23–448.28)	0.0 (0.0–0.23)	<0.001
STS-PROM, %	2.76 (1.97-5.10)	2.61 (1.83-4.50)	0.034
THV type			0.048
Balloon-expandable	36 (9.8)	9 (4.8)	-
Self-expanding	330 (90.2)	177 (95.2)	-
Prosthesis brand			-
Venus-A	231 (64.2)	96 (51.6)	-
Taurus	42 (11.5)	6 (3.2)	-
VitaFlow	21 (5.7)	14 (7.5)	-
Evolut	22 (6.0)	1 (0.5)	-
ProStyle	9 (2.5)	0	-
Venus+Numed	1 (0.3)	51 (27.4)	-
TaurusTrio	0	8 (4.3)	-

Data are n (%) or median (IQR). Continuous variables: Mann–Whitney U test; categorical variables: Fisher exact test (Monte Carlo estimation). BAV subtype percentages use all BAV cases.

Table S6

B. Sensitivity analyses with additional device and STS adjustment

Analysis	N / deaths	+ Device type HR (95% CI)	P value	+ Device type and STS HR (95% CI)	P value
Baseline RDW-CV	1369 / 196	1.17 (1.02–1.33)	0.024	1.17 (1.03–1.34)	0.019
3-month RDW-CV change	652 / 65	1.51 (1.19–1.92)	<0.001	1.51 (1.19–1.93)	<0.001
6-month RDW-CV change	620 / 77	1.37 (1.08–1.73)	0.009	1.38 (1.09–1.74)	0.008
6-month change × BAV	617 / 77	2.12 (1.31–3.44)	0.002	2.10 (1.30–3.40)	0.002

Models adjust for age, sex, AS/AR phenotype, eGFR, hemoglobin, and MCV; change models also include baseline RDW-CV. Additional covariates are device type (balloon- vs self-expanding) and STS, as indicated. HRs are per 1-SD increase; the final row reports the interaction term. AS, aortic stenosis; AR, aortic regurgitation; BAV, bicuspid aortic valve; TAV, tricuspid aortic valve; HR, hazard ratio; CI, confidence interval; IQR, interquartile range; STS, Society of Thoracic Surgeons; THV, transcatheter heart valve.

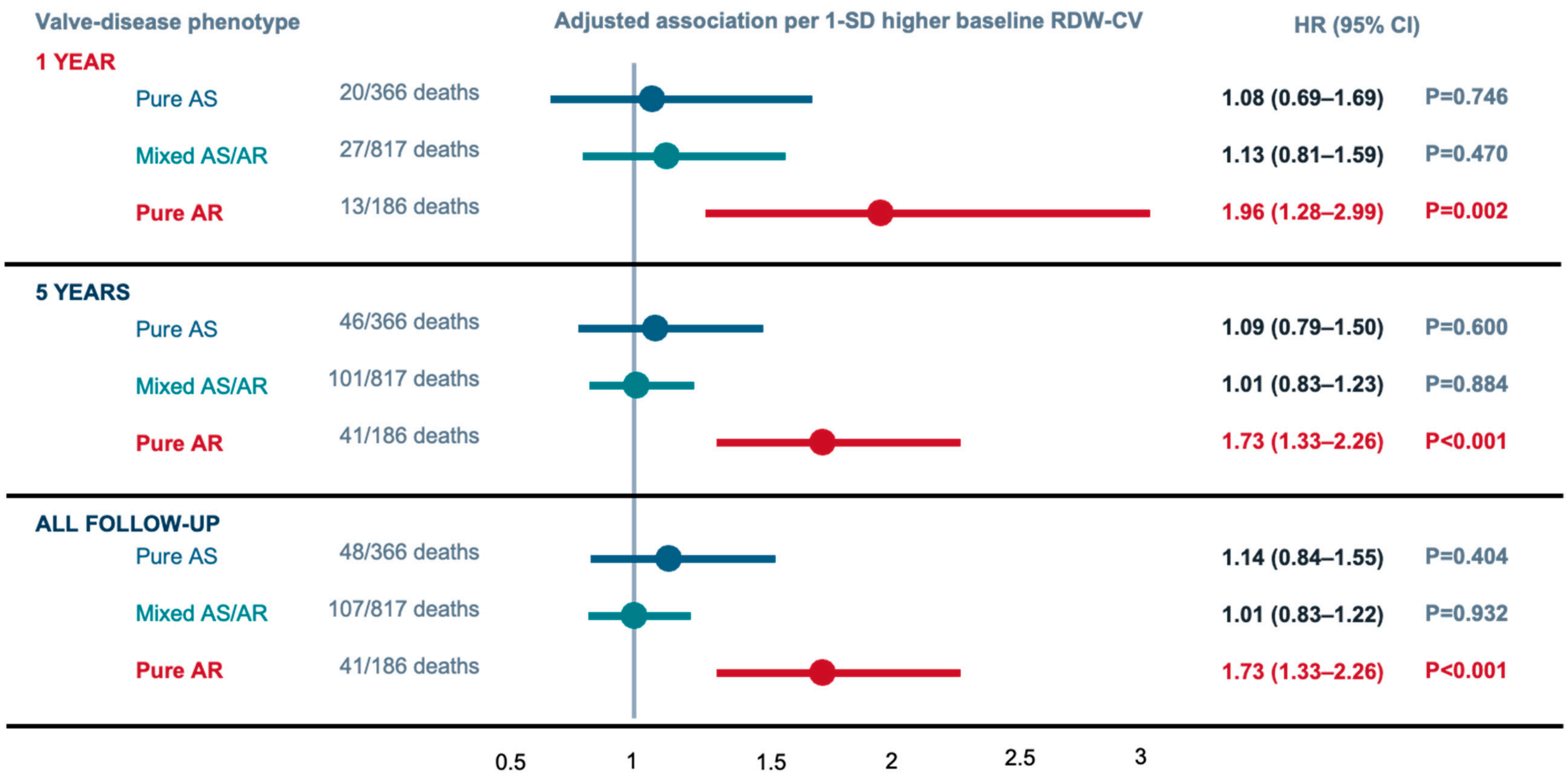

Figure S1. Phenotype-specific associations between baseline RDW-CV and mortality. Adjusted mortality associations per 1-SD higher baseline RDW-CV in pure AS, mixed AS/AR, and pure AR at 1 year, 5 years, and over all follow-up, with interaction p values.

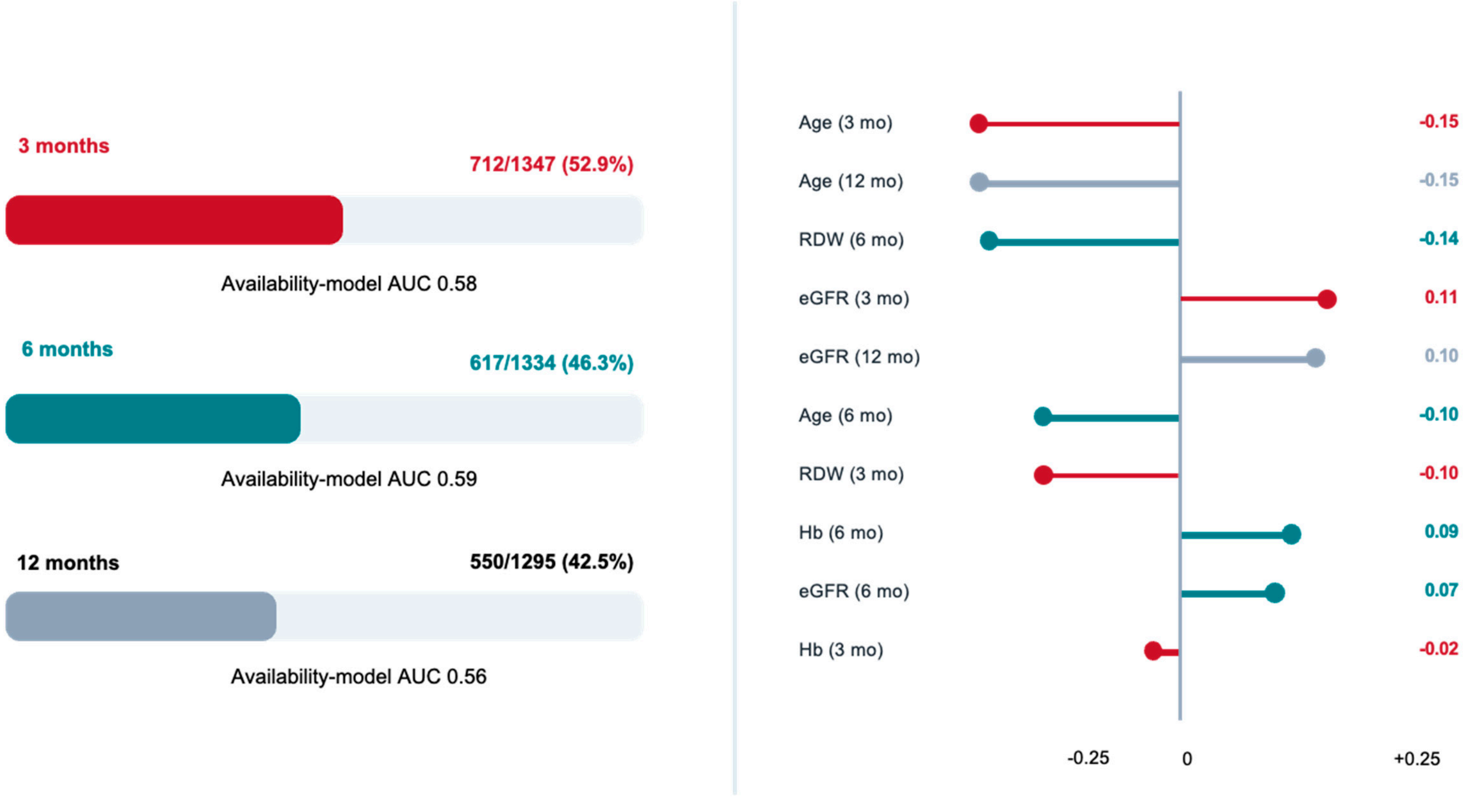

Figure S2. Availability of RDW-CV measurements at 3, 6, and 12 months and standardized differences between patients with and without measurements..
